# Supplementary material for: DAP5 enables main ORF translation on mRNAs with structured and uORF-containing 5′ leaders
Source: Nat Commun. 2022 Dec 6;13:7510. doi: 10.1038/s41467-022-35019-5 (PMC9726905; doi:10.1038/s41467-022-35019-5)
Supplement: Supplementary file 1 — Supplementary Information [file 41467_2022_35019_MOESM1_ESM.pdf]

Supplementary Information

DAP5 enables main ORF translation on mRNAs with structured and uORF-containing 5´ leaders

Ramona Weber[1,2,*], Leon Kleemann[1,3], Insa Hirschberg[4], Min-Yi Chung[1], Eugene Valkov[1,5], and Cátia Igreja[1,*]

*Correspondence to: ramona.weber@uzh.ch (R.W.), catia.igreja@tuebingen.mpg.de (C.I.)

Weber *et al.* Supplementary Fig. 1

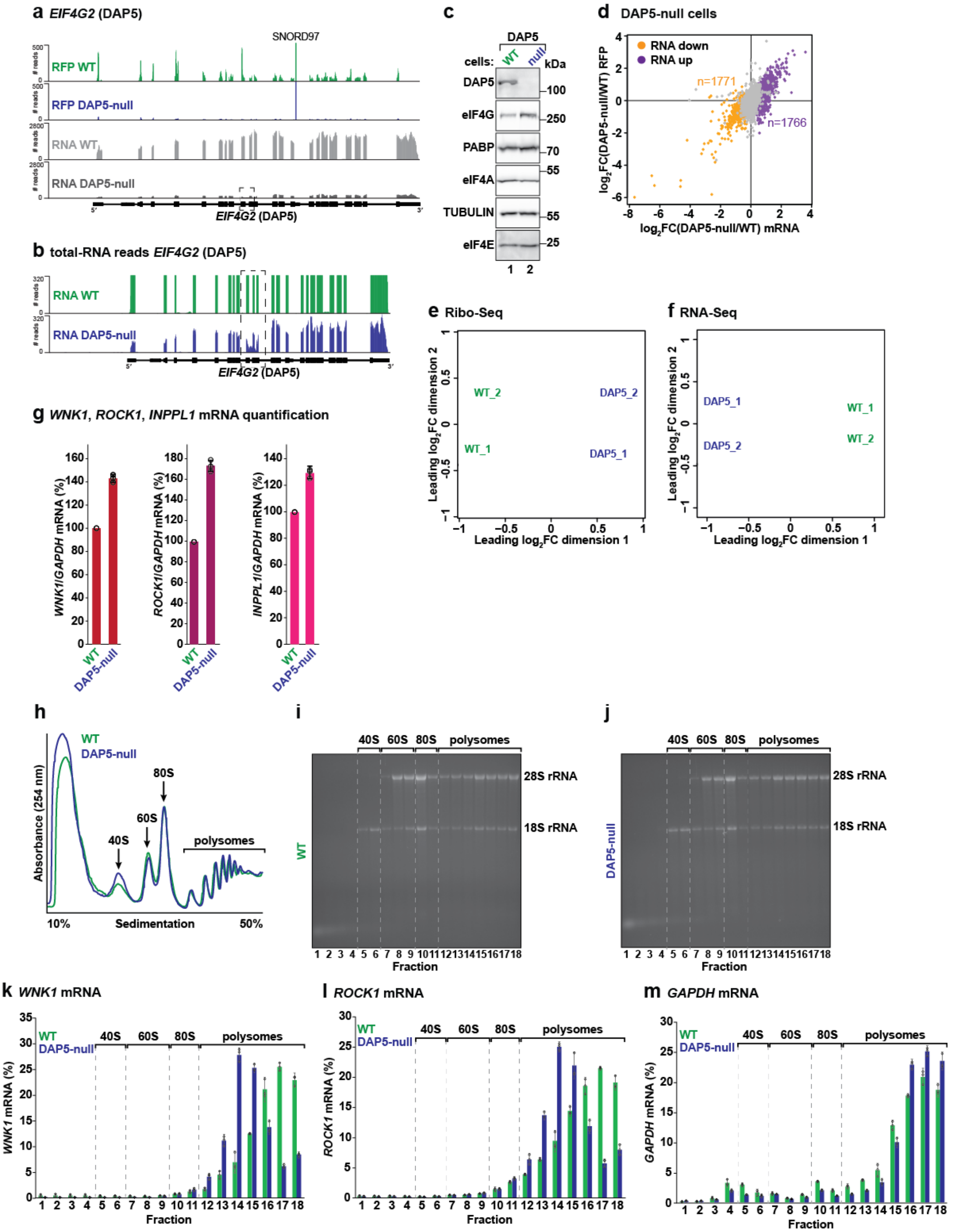

Supplementary Fig. 1 (related to Fig. 1). Characterization of DAP5-null cells

(a, b) Ribosome footprints and total mRNA reads distribution along *WNK1* mRNA in wild type (WT, green) and DAP5-null cells. Of note, RFP and total RNA counts for *WNK1* are

drastically reduced in the null cells. Dashed box indicates the position of the CRISPR-Cas9 edited region. In panel b, read counts scale for total RNA in DAP5-null cells was changed to show the presence of reads in intron 9 as a result of genome editing. SNORD97 (small nucleolar RNA, C/D Box 97) is encoded in intron 13 of *DAP5*.

(c) Western blot demonstrating the loss of DAP5 expression in the null cells. The expression of eIF4E, eIF4G and eIF4A is not decreased in the absence of DAP5. eIF4G and PABP protein levels are even increased in the null cells. TUBULIN served as a loading control. TUBULIN antibody recognizes an epitope common among the α-tubulin subunits.

(d) Comparative analysis of translation efficiency (TE) in wild type (WT) and DAP5-null HEK293T cells as described in Fig. 1a. Genes with increased (n=1771 genes) and decreased (n=1766 genes) mRNA abundance are highlighted in purple and orange, respectively.

(e, f) Multidimensional scaling (MDS) analysis for the Ribo-Seq (e) and RNA-Seq (f) replicate libraries from HEK293T wild type (WT) and DAP5-null cells. The Ribo-Seq and RNA-Seq experiments were reproducible as replicates clustered together.

(g) Quantification of *WNK1*, *ROCK1* and *INPPL1* mRNA levels by quantitative qPCR following reverse transcription. *GAPDH* mRNA levels served as normalization control. Values were set to 100% in WT cells. The mean values +/- SD of three independent experiments are shown.

(h) UV absorbance profile at 254 nm of HEK293T WT (green) and DAP5-null cells (blue) cell extracts after polysome sedimentation in a sucrose gradient. Absorbance peaks at 254 nm representing free 40S and 60S subunits, 80S monosomes and polysomes are indicated. With the exception of a reproducible increase in the free 40S subunits peak, WT and DAP5-null cells polysome profiles after sucrose density gradient separation were similar.

(i, j) Ethidium bromide staining of total RNA extracted from the different sucrose fractions. Due to high abundance, 28S and 18S rRNAs positions in the gel are readily detected.

(k-m) Abundance profiles for *WNK1* (k), *ROCK1* (l) and *GAPDH* (m) mRNAs across the density gradient in WT (green) and DAP5-null (blue) cells. mRNA abundance was determined by quantitative PCR (qPCR). Bars represent the mean value; error bars represent standard deviations (SD) of *WNK1*, *ROCK1* and *GAPDH* mRNA levels in each fraction (n=3 biologically independent experiments). Source data are provided as a Source Data file.

Weber *et al.* Supplementary Fig. 2

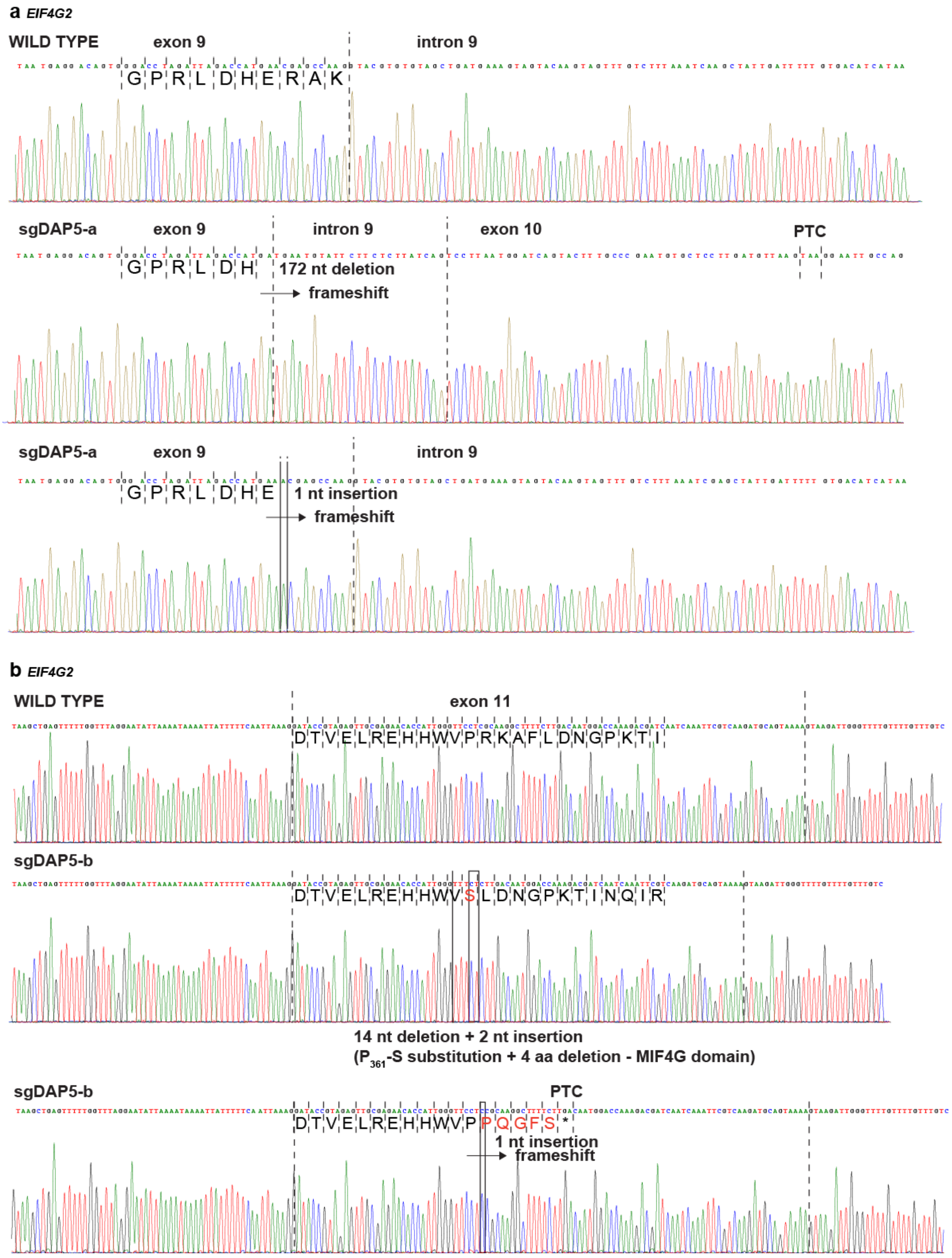

Supplementary Fig. 2 (related to Fig. 1). Characterization of DAP5-null cells

(a, b) Sanger sequencing of DAP5 genomic region targeted by the sgRNA DAP5-a and DAP5-b. Frameshift mutations were detected in exons 10 and 12. These generate premature STOP codons (PTC) and deletions in *DAP5*.

Weber *et al.* Supplementary Fig. 3

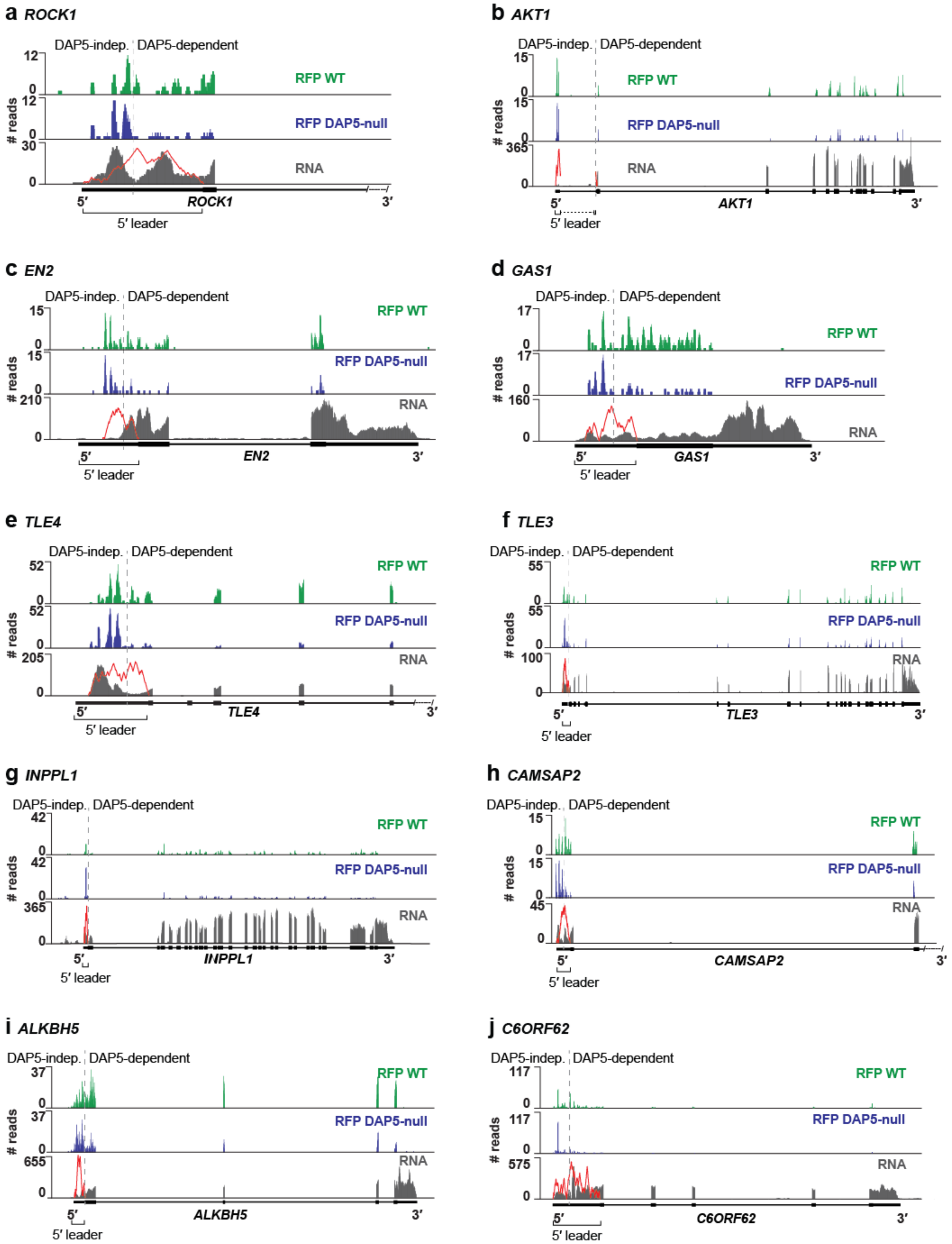

Supplementary Fig. 3 (related to Fig. 1). Ribosome densities and mRNA read counts in DAP5 targets

(a-j) Ribosome footprints and total mRNA reads distribution along the sequence of different DAP5 target mRNAs in wild type (WT, green) and DAP5-null (blue) cells. The predicted propensity for secondary structure across the 5′ leaders is illustrated in red. Gene annotation is depicted below the profiles. DAP5-independent (indep.) and -dependent translation is indicated with a grey dashed line.

Weber *et al.* Supplementary Fig. 4

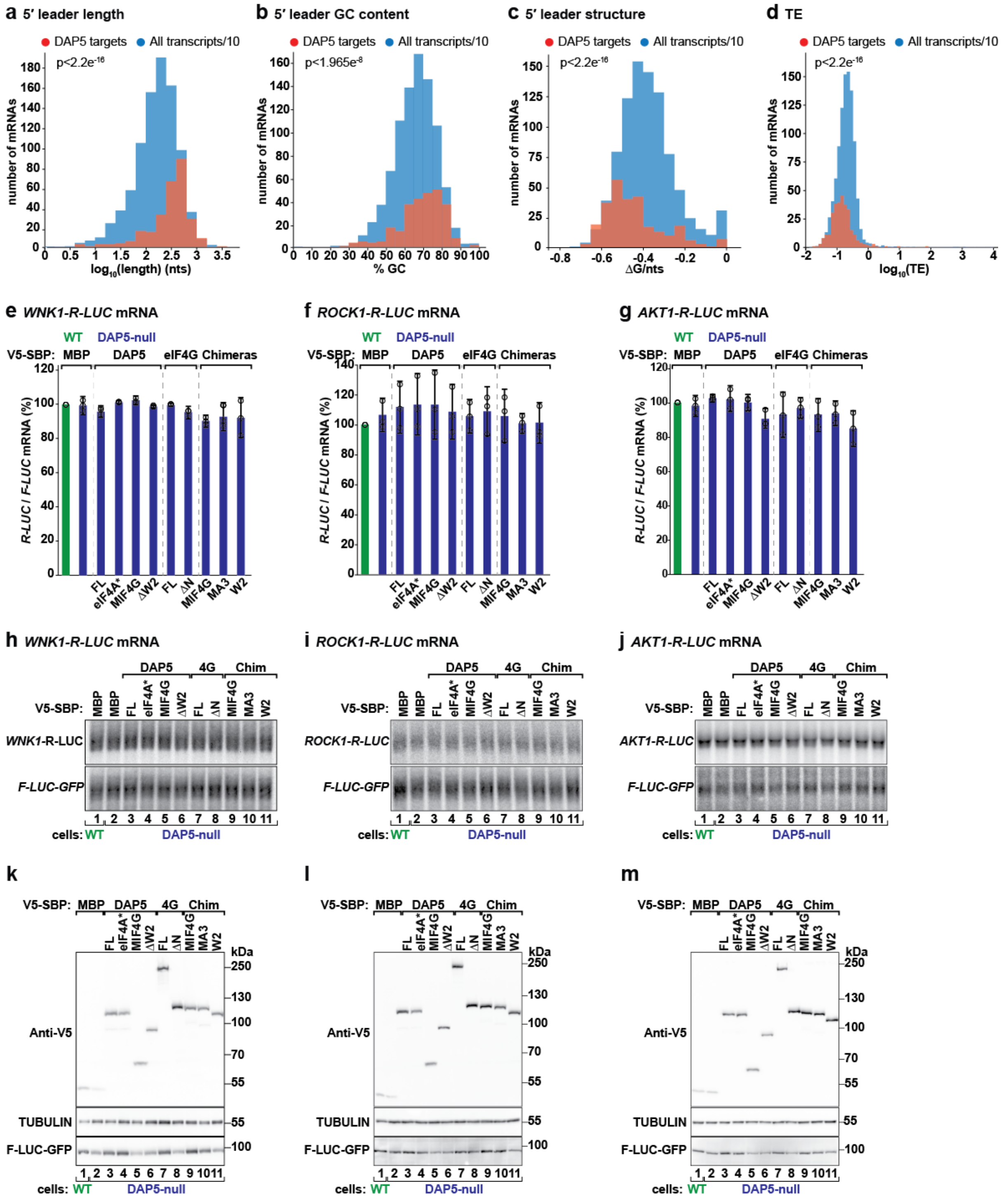

Supplementary Fig. 4 (related to Figs. 1 and 2). DAP5-dependent translation occurs in mRNAs with structured 5′ leaders

(a-c) The histograms show the distribution of length ($\log_{10}$ nts), GC content (%) and minimum free energy (ΔG/ nts) of the 5′ leaders in DAP5 targets (red) and all transcripts expressed in

HEK293T cells (blue). The number of all transcripts is reduced by a factor of 10 for visualization purposes. Statistical significance was calculated with the one-sided Wilcoxon rank sum test. Bin width is 0.2 in a, 5% in b and 0.5 in c.

(d) Histogram depicting the range of TE (log_{10}) of the DAP5 targets (red) and all other mRNAs expressed in HEK293T cells (blue). DAP5 targets show lower TE than all other transcripts ($p<2.2e^{-16}$). Statistical significance was calculated using the one-sided Wilcoxon rank sum test. Bin width=0.1.

(e-j) WT and DAP5-null cells were transfected with plasmids expressing *WNK1*-, *ROCK1*- or *AKT1*-R-LUC, V5-SBP-MBP, V5-SBP-DAP5 (FL or mutants), V5-SBP-eIF4G (FL or ΔN) or V5-SBP-Chimeras. *R-LUC* mRNA levels were determined by northern blotting, normalized to *F-LUC-GFP* and set to 100% in WT cells. Bars represent the mean value; error bars represent SD (n=3 biologically independent experiments). Representative northern blots are shown in h-j.

(k-m) Immunoblots depicting the expression of the proteins used in Fig. 2a-c. Source data are provided as a Source Data file.

Weber *et al.* Supplementary Fig. 5

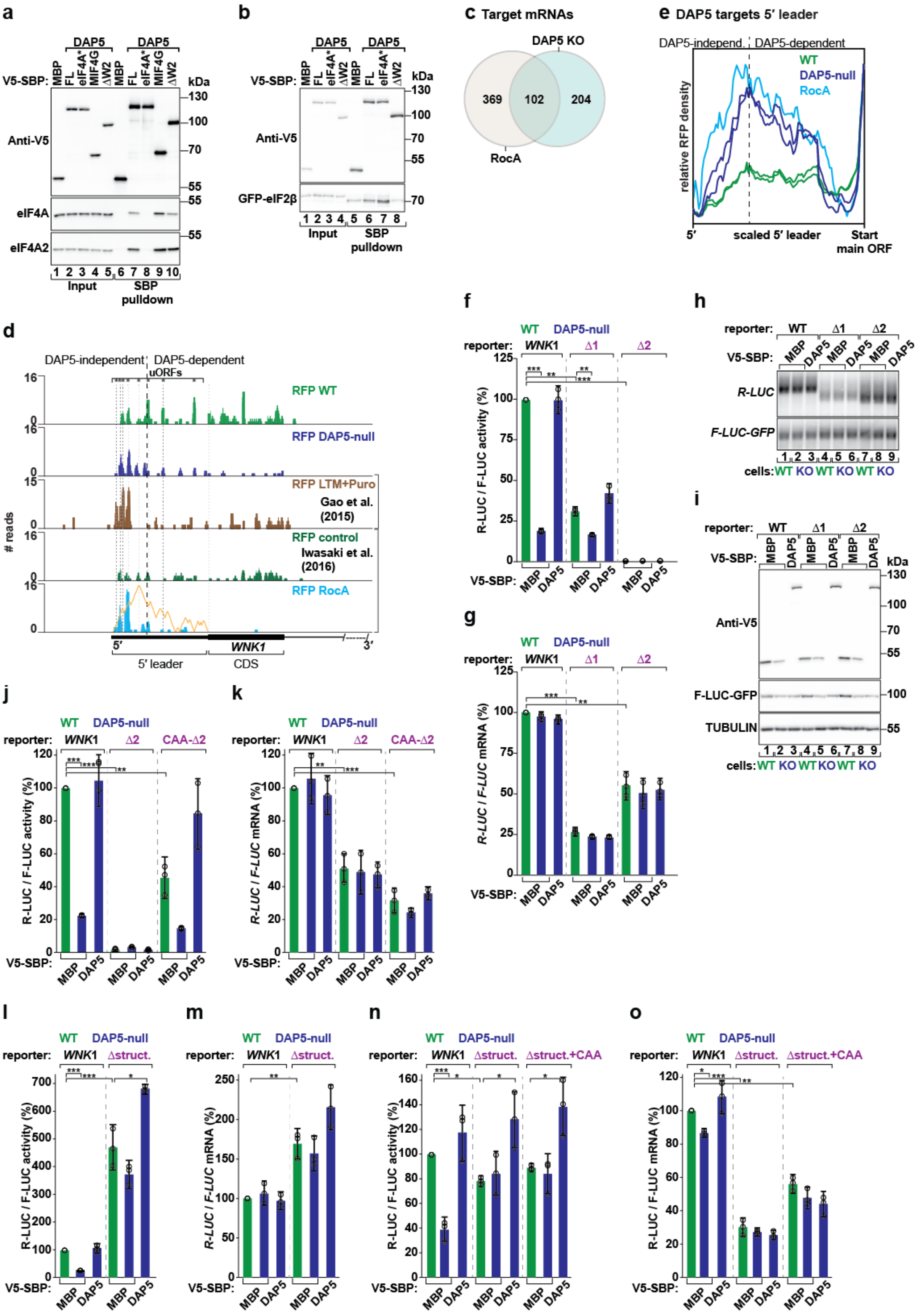

Supplementary Fig. 5 (related to Figs. 2 and 3). DAP5-dependent translation requires the RNA helicase eIF4A

(a, b) Streptavidin-binding protein (SBP) affinity pulldowns were performed two days post cellular transfection with SBP-V5-MBP or V5-SBP-DAP5 (FL or mutants) and GFP-eIF2β (b). Input (1% for the V5 proteins, 0.3% for eIF4A, eIF4A2 and GFP-eIF2β) and pulldown fractions (1% for the V5 proteins, 2% for eIF4A, eIF4A2 and GFP-eIF2β) were analysed by western blotting with anti-V5, eIF4A, eIF4A2 and GFP antibodies.

(c) Venn diagram showing the number of common (n=102 genes; $p=5.1995e^{-97}$ using a one-sided hypergeometric test) and unique genes with decreased TE in DAP5 knockout (KO) HEK293T cells and HEK293 cells treated with 0.003 μM of Rocaglamide A (RocA)[1].

(d) Ribosome footprints and total mRNA reads distribution along *WNK1* exon 1 including the 5′ leader and the most 5′ proximal coding sequence in WT (green) and DAP5-null (blue) cells. Also shown are the ribosome footprint profiles (RFPs) in HEK293 cells treated with lactimidomycin (LTM) and puromycin (Puro, brown) obtained by Gao and co-workers[2] and in HEK293 cells upon treatment with 0.003 μM of RocA[1] (cyan). The predicted propensity for secondary structure across *WNK1* 5′ leader is illustrated in orange. uORFs position in the 5′ leader is indicated with the corresponding start codons and asterisks. Gene annotation is depicted below the profiles. DAP5-independent and -dependent translation is indicated with a black dashed line. CDS: coding sequence.

(e) Metagene analysis of ribosome density for the 5′ leaders of the DAP5 targets (n=306 genes) in WT (green), DAP5-null (dark blue), and RocA-treated cells (light blue)[1]. Ribosome densities were determined as the ratio of footprints within the 5′ leader relative to the footprints at the annotated downstream CDS start codon. The black dashed line indicating DAP5-independent (indep.) and DAP5-dependent translation was defined as the position along the 5′ leaders in which RFP density decreases in the absence of DAP5.

(f-m) WT and DAP5-null cells were transfected with plasmids expressing *WNK1*-R-LUC reporters, V5-SBP-MBP or V5-SBP-DAP5. Following transfection, luciferase activities were measured (f, j, l) and mRNA levels determined by northern blotting or qPCR (g, h, k, m). R-LUC activity and mRNA levels were normalized to the transfection control F-LUC-GFP and set to 100% in WT cells. Bars show the mean value and error bars indicate the SD (n=3 biologically independent experiments). Significance was determined by One-way ANOVA test and indicated significant if $p<0.05$ (*), $p<0.005$ (**) and $p<5e^{-5}$ (***). A representative northern blot is shown in h. (i) The immunoblot shows the expression levels of the proteins used in the assay depicted in Fig. 3f. Membranes were incubated with anti-V5, GFP and TUBULIN. Source data are provided as a Source Data file.

Weber *et al.* Supplementary Fig. 6

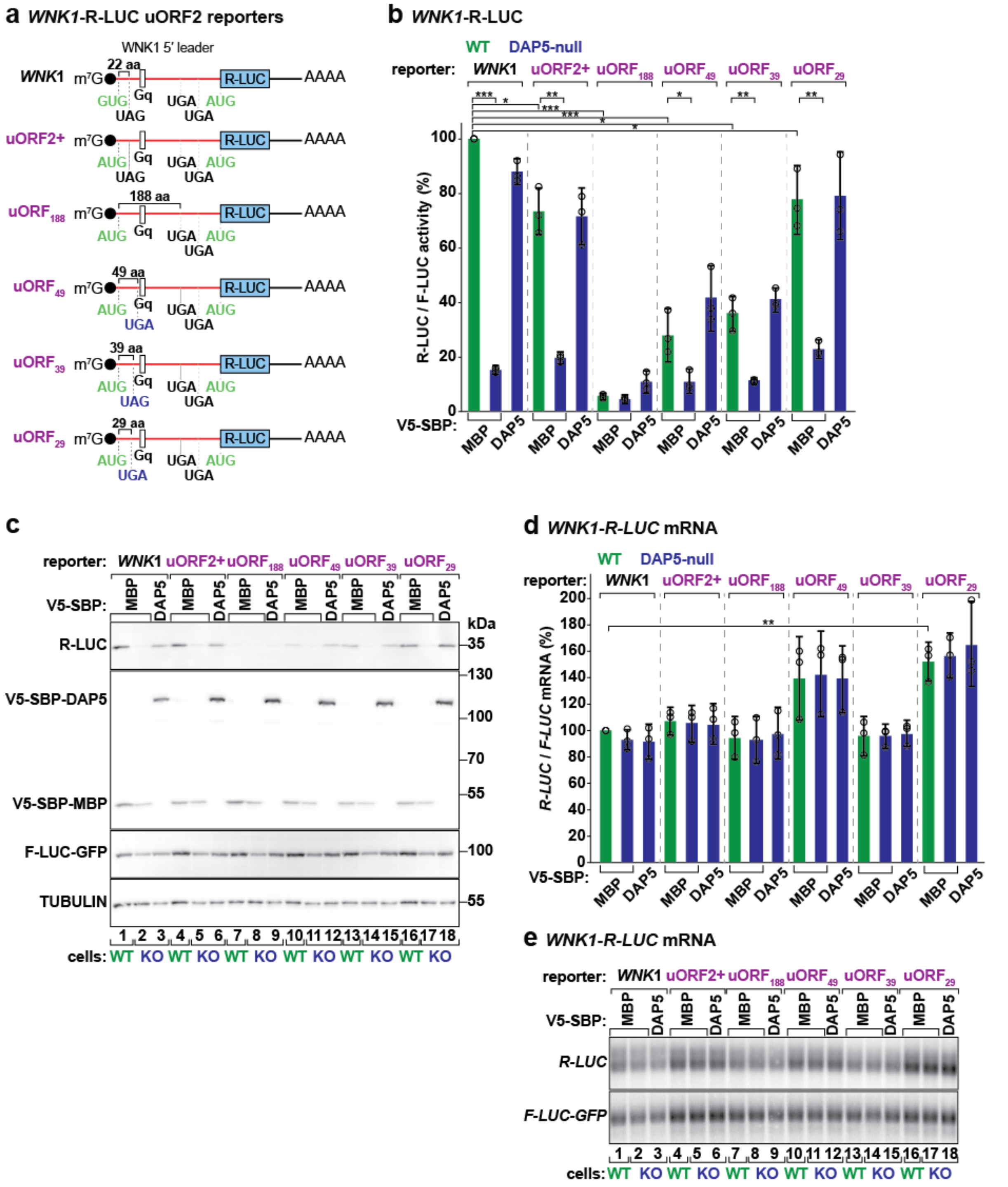

Supplementary Fig. 6 (related to Figs. 4 and 6). Short uORFs support DAP5-dependent translation

(a) Schematic representations of the *WNK1*-R-LUC reporters with changes in uORF2 initiation context and length. uORF2 GUG is in frame with the R-LUC and is 22 codons long. Three

STOP codons can be found downstream and in frame with uORF2 GUG. uORF2+: GUG start codon was substituted by AUG to favour the initiation of translation. $uORF_{188}$: first STOP codon in frame with uAUG was removed; uORF is then 188 codons long. $uORF_{49}$, $uORF_{39}$, $uORF_{29}$: position of the STOP codon was moved to 49, 39 or 29 codons downstream of uAUG, respectively. aa: amino acids.

(b-e) WT and DAP5-null cells were transfected with different *WNK1*-R-LUC reporters, F-LUC-GFP and V5-SBP-MBP or V5-SBP-DAP5. Following transfection, luciferase activities (b) and expression (c) were measured and mRNA levels determined by northern blotting (d, e). R-LUC values were normalized to the transfection control F-LUC-GFP. The graphs show the protein (b) and mRNA levels (d) in WT and null cells, set to 100% in WT cells expressing *WNK1*-R-LUC. Bars indicate the mean value; error bars represent SD (n=3 biologically independent experiments). Significance was determined using One-sided ANOVA test and indicated significant if $p<0.05$ (*), $p<0.005$ (**) and $p<5e^{-5}$ (***). The immunoblot showing the expression of the different proteins is depicted in panel c. TUBULIN served as a loading control. A representative northern blot is shown in e. Source data are provided as a Source Data file.

Weber *et al.* Supplementary Fig. 7

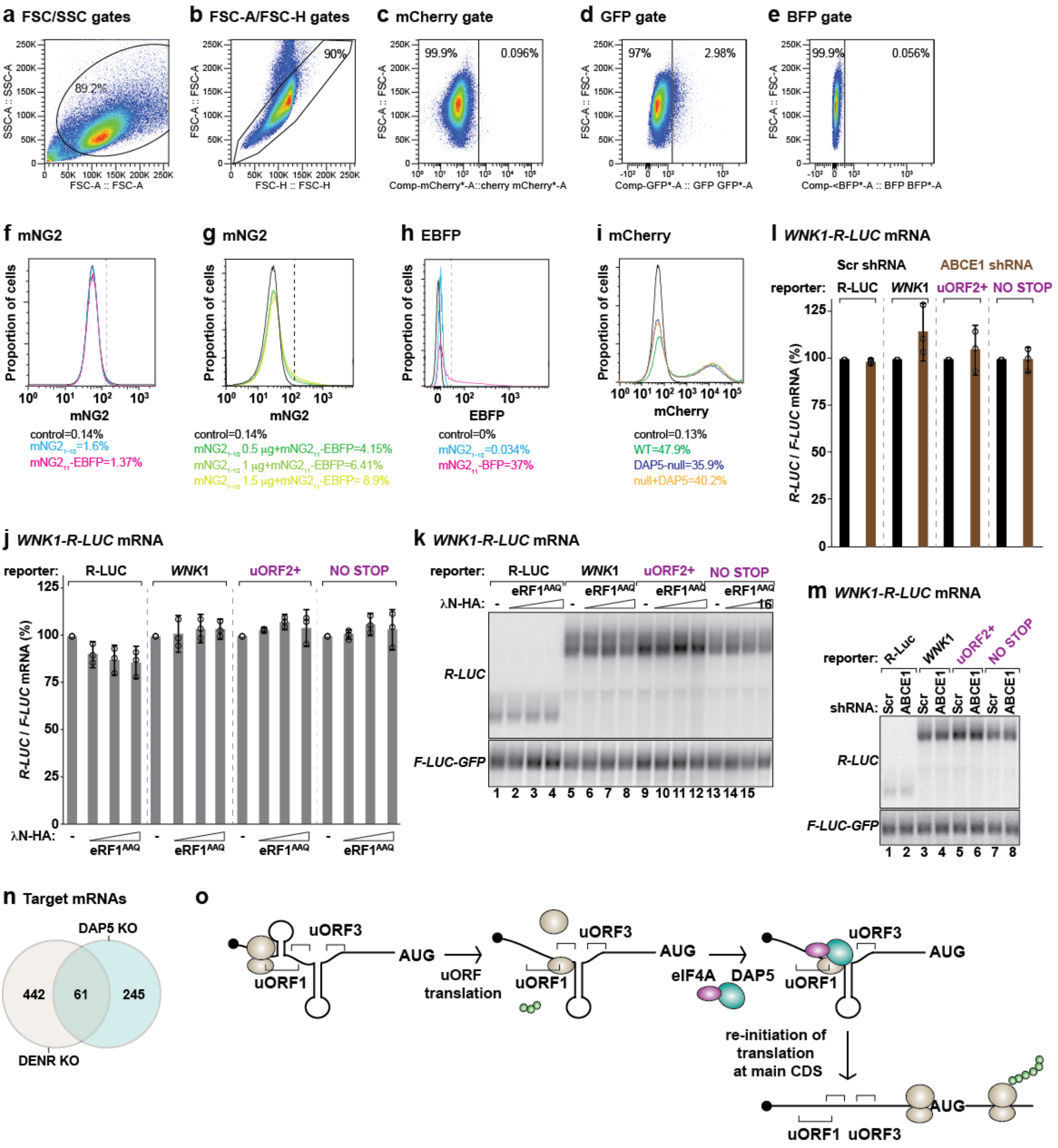

Supplementary Fig. 7 (related to Figs. 6 and 7). Detection of uORF translation in DAP5 targets

(a-e) Gating strategy applied in the flow cytometry analysis of HEK293T cells expressing the split-fluorescent reporter. Preliminary forward vs side scatter (FSC vs SSC) gating was applied to unstained cells to identify the cells of interest based on size and complexity. Following this selection, doublets were excluded with forward scatter height (FSC-H) versus forward scatter

area (FSC-A) gating. Singlet gates were then established for the mCherry, GFP and BFP fluorophores. The boundaries between “positive” and “negative” staining cell populations were defined using unstained control cells.

(f, g) Histograms of the mNG2 fluorescence, quantified by flow cytometry, in non-transfected (control, black trace) or HEK293T cells expressing $mNG2_{1\text{-}10}$ (light blue trace), $mNG2_{11}$-EBFP (pink trace), or increasing amounts of $mNG2_{1\text{-}10}$ (green-yellow traces) and $mNG2_{11}$-EBFP. mNG2 expression is plotted on a log scale and represents around $2x10^5$ cells.

(h) Histograms of the EBFP fluorescence, quantified by flow cytometry, in non-transfected (control, black trace) or HEK293T cells expressing $mNG2_{1\text{-}10}$ (light blue trace) or $mNG2_{11}$-BFP (pink trace). EBFP expression is plotted on a log scale and represents around $2x10^5$ cells.

(i) Histograms of the mCherry fluorescence, quantified by flow cytometry, in non-transfected cells (control, black trace), and WT (green trace) or DAP5-null HEK293T cells expressing mCherry, V5-SBP-MBP (blue trace) or V5-SBP-DAP5 (yellow trace). mCherry expression is plotted on a log scale and represents around $2x10^5$ cells.

(j, k) WT cells expressing increasing concentrations of $eRF1^{AAQ}$ were transfected with different *WNK1*-R-LUC reporters and F-LUC-GFP. Following transfection, luciferase mRNA levels were determined by northern blotting. R-LUC values were normalized to the transfection control F-LUC-GFP. The graph shows the luciferase mRNA levels in cells and set to 100% in the absence of $eRF1^{AAQ}$. Bars indicate the mean value; error bars represent SD (n=3 biologically independent experiments). A representative northern blot is present in panel f. See also Fig. 7.

(l, m) Scramble (Scr, balck) and ABCE1 (brown) shRNA-treated cells were transfected with different *WNK1*-R-LUC reporters and F-LUC-GFP. Following transfection, luciferase mRNA levels were determined by northern blotting. R-LUC values were normalized to the

transfection control F-LUC-GFP and set to 100% in control knockdown cells. A representative northern blot is present in panel m. See also Fig. 7.

(n) Venn diagram showing the number of common (n=61 mRNAs; $p=1.2985e^{-21}$ using a one-tailed hypergeometric test) and unique genes with decreased TE in DAP5 knockout (KO) HEK293T cells and DENR KO HeLa cells[3].

(o) DAP5 drives re-initiation following uORF translation. Simplified schematics of the role of DAP5 in translation. DAP5 target mRNAs contain structured regions (indicated as stem loops) and multiple uORFs (indicated as brackets) that initiate with near-cognate start codons. Secondary structures at the 5′ leaders stall scanning PICs and trigger uORF translation. The non-optimal initiation contexts of the uORFs may result in initiation at different near-cognate start codons, as the PICs bypass some but not all uORFs (leaky scanning). Post-termination translation complexes at the STOP codon of uORFs are recognised by the DAP5 (in cyan)-eIF4A (in purple) complex which then fuels a new cycle of scanning and translation at a downstream start codon. Multiple cycles of re-initiation move the ribosome (in brown) through the burdened 5′ leaders towards the main CDS AUG. Green circles represent peptide chains. Black dot indicates the cap structure of the mRNA. Source data are provided as a Source Data file.

Supplementary Table 4. List of DNA constructs used in this study

Recombinant DNA	Source	
pT7-V5-SBP-C1-MBP	This study	N/A
pT7-V5-SBP- C1-*Hs*DAP5	This study	Uniprot: P78344-1
pT7-V5-SBP- C1-*Hs*DAP5 L85A L88A F93A (eIF4A*)	This study	N/A
pT7-V5-SBP- C1-*Hs*DAP5 1-475 (MIF4G)	This study	N/A
pT7-V5-SBP- C1-*Hs*DAP5 1-722 (ΔW2)	This study	N/A
pT7-V5-SBP- C1-*Hs*eIF4G	This study	Uniprot: Q04637-9
pT7-V5-SBP- C1-*Hs*eIF4G 682-1606 (ΔN)	This study	N/A
pT7-V5-SBP- C1-*Hs*DAP5-MIF4G (4G) chimera	This study	N/A
pT7-V5-SBP- C1-*Hs*DAP5-MA3 (4G) chimera	This study	N/A
pT7-V5-SBP- C1-*Hs*DAP5-W2 (4G) chimera	This study	N/A
pCIneo-R-LUC	[4]	N/A
pCIneo-*WNK1*-R-LUC	This study	N/A
pCIneo-*WNK1*-R-LUC Δ1 (Δ1-217)	This study	N/A
pCIneo-*WNK1*-R-LUC Δ2 (Δ1-266)	This study	N/A
pCIneo-*WNK1*-R-LUC CAA-Δ2 (Δ1-266 replaced by 18x CAA)	This study	N/A
pCIneo-*WNK1*-R-LUC Δstruct (Δ199-576)	This study	N/A
pCIneo-*WNK1*-R-LUC Δstruct+CAA (Δ199-576 replaced by 18x CAA)	This study	N/A
pCIneo-*WNK1*-R-LUC uORF2+	This study	N/A
pCIneo-*WNK1*-R-LUC uORF188	This study	N/A
pCIneo-*WNK1*-R-LUC NO STOP	This study	N/A
pCIneo-*WNK1*-R-LUC uORF 49	This study	N/A
pCIneo-*WNK1*-R-LUC uORF 39	This study	N/A
pCIneo-*WNK1*-R-LUC uORF 29	This study	N/A
pCIneo-*WNK1*-R-LUC uORF118	This study	N/A
pCIneo-*WNK1*-R-LUC uORF30	This study	N/A
pCIneo-*WNK1*-R-LUC uORF19	This study	N/A
pCIneo-*WNK1*-R-LUC uORF9	This study	N/A
pCIneo-*WNK1*-R-LUC ΔSTART	This study	N/A
pCIneo-*WNK1*-R-LUC ΔSTOP	This study	N/A
pCIneo-*WNK1*-R-LUC ΔSTART/STOP		
pCIneo-*ROCK1*-R-LUC	This study	N/A
pCIneo-*AKT1*-R-LUC	This study	N/A
pEGFP-N3-F-LUC	[5]	N/A
pT7-EGFP-C1-MBP	[5]	N/A
pT7-EGFP-C1-4EBP-Chimera	[6]	N/A
pSFFV_mNG2(11)1-10	[7]	Addgene_82610
pcDNA3.1-MCS-mCherry	This study	N/A
EBFP-N1	a gift from Michael Davidson	Addgene_54595
pcDNA3.1-mNG2(1-10)	This study	N/A
pCIneo-*WNK1*-mNG2(11)-EBFP	This study	N/A
pCIneo-*WNK1*-uORF188-mNG2(11)-EBFP	This study	N/A
pλN-HA-C1-eRF1 G183A G184A (AAQ)	This study	Uniprot: P62495-1
pT7-EGFP-C1-eIF2β	This study	Uniprot: P20042-1
pSpCas9(BB)-2A-Puro (PX459)	[8]	Addgene 48139
pSUPERpuro-BglII-scrambled	[9]	N/A
pSUPERpuro-BglII-*Hs*ABCE1 (1+2)	This study	N/A

Supplementary Table 5. Primers used in this study

		sequence (5' to 3')
**qPCR**		
WNK1	fwd	CAGGGTCAGCCATCCTCAAGTA
	rev	GTGTCGCAACTGGGATCTGAGG
ROCK1	fwd	CCCCTCGAACGCTTTCTACAAG
	rev	ATCTACCTGTAGGCAAACCCGC
GAPDH	fwd	CTCTGCTCCTCCTGTTCGACAG
	rev	TTCCCGTTCTCAGCCTTGACGG
INPPL1	fwd	CAGGTTGAAGCCAGAGGGAGCT
	rev	GGTGGAGGTGGAAAGTCTGGAG
**sgRNA**		
sgDAP5-a		CACGTACCTTGGCTCGTTCA
sgDAP5-b		ACACCATTGGGTTCCTCGCA
gDNA-locus-a1		GAGTTAGAACTGATCAACCAGA
gDNA-locus-a1		CCAGTATTACCTGCAGCAGGAA
gDNA-locus-a2		AGAAAGCCCTATACTAGATTCT
gDNA-locus-a2		TTCTCGCAACTCTACGGTATCC
gDNA-locus-b		TTCCTGCTGCAGGTAATACTGG
gDNA-locus-b		ACTTCTTCCAAAAATGGCAGAC
**shRNA**		
Scramble		ATTCTCCGAACGTGTCACG[9]
ABCE1-1		GCTACAGCGAGTACGTTTACCT[10]
ABCE1-2		CCGTGGATCTGAATTACAATT[11]
**Ribosome profiling**		
30 nt RNA marker		AUGUACACGGAGUCGAGCUCAACCCGCAAC-P
27 nt RNA marker		AUGUACACGGAGUCGAGCUCAACCCGC-P
3' adapter		rApp/NNNNTGGAATTCTCGGGTGCCAAGG/3InvdT/
5' adapter (RNA)		GUUCAGAGUUCUACAGUCCGACGAUCNNNN
Reverse transcription primer		GCCTTGGCACCCGAGAATTCCA
Forward primer		AATGATACGGCGACCACCGAGATCTACACGTTCAGAGTT CTACAGTCCGA
Barcoded reverse primer		CAAGCAGAAGACGGCATACGAGATNNNNNNGTGACTGG AGTTCCTTGGCACCCGAGAATTCCA

Supplementary Table 6. Antibodies used in this study

Antibodies	Source	Identifier	Dilution
Mouse monoclonal anti-GFP	Roche	Cat. #11814460001	1:3000
Rabbit polyclonal anti-*Hs*WNK1	Cell Signaling Technology	Cat. #4979	1:1000
Rabbit polyclonal anti-*Hs*ROCK1	Abcam	Cat. #ab97592	1:1000
Mouse monoclonal anti-*Hs*SHIP2 (INPPL1)	Santa Cruz Biotechnology	Cat. #sc-166641	1:1000
Rabbit polyclonal anti-*Hs*DAP5	Bethyl laboratories	Cat. #A302-249A-M	1:1000
Rabbit polyclonal anti-*Hs*eIF4A	Abcam	Cat. #ab31217	1:1000
Rabbit polyclonal anti-*Hs*eIF4A2	Abcam	Cat. #ab31218	1:1000
Mouse monoclonal anti-HA (HRP)	Roche	Cat. #12013819001	1:5000
Mouse monoclonal anti-TUBULIN	Sigma Aldrich	Cat. #T6199	1:1000
Mouse monoclonal anti-V5	LSBio LifeSpan BioSciences, Inc.	Cat. #LS-C57305	1:5000
Mouse monoclonal anti-*Renilla* luciferase	Abcam	Cat. #ab185925	1:1000
Rabbit polyclonal anti-*Hs*ABCE1	Abcam	Cat. #ab32270	1:1000
Rabbit polyclonal anti-*Hs*eIF4E	Bethyl laboratories	Cat. #A301-154A	1:1000
Rabbit polyclonal anti-*Hs*eIF4G	Bethyl laboratories	Cat. #A301-776A	1:1000
Rabbit polyclonal anti-*Hs*PABP	Abcam	Cat. #ab21060	1:3000
Donkey polyclonal anti-rabbit IgG (HRP)	GE Healthcare	Cat. #NA934V	1:10 000
Sheep polyclonal anti-mouse IgG (HRP)	GE Healthcare	Cat. #RPN4201	1:10 000

References:

1 Iwasaki, S., Floor, S. N. & Ingolia, N. T. Rocaglates convert DEAD-box protein eIF4A into a sequence-selective translational repressor. *Nature* **534**, 558-561, doi:10.1038/nature17978 (2016).

2 Gao, X. *et al.* Quantitative profiling of initiating ribosomes in vivo. *Nat Methods* **12**, 147-153, doi:10.1038/nmeth.3208 (2015).

3 Bohlen, J. *et al.* DENR promotes translation reinitiation via ribosome recycling to drive expression of oncogenes including ATF4. *Nat Commun* **11**, 4676, doi:10.1038/s41467-020-18452-2 (2020).

4 Pillai, R. S., Artus, C. G. & Filipowicz, W. Tethering of human Ago proteins to mRNA mimics the miRNA-mediated repression of protein synthesis. *Rna* **10**, 1518-1525, doi:10.1261/rna.7131604 (2004).

5 Lazzaretti, D., Tournier, I. & Izaurralde, E. The C-terminal domains of human TNRC6A, TNRC6B, and TNRC6C silence bound transcripts independently of Argonaute proteins. *Rna* **15**, 1059-1066, doi:10.1261/rna.1606309 (2009).

6 Peter, D. *et al.* Molecular Architecture of 4E-BP Translational Inhibitors Bound to eIF4E. *Mol Cell*, doi:10.1016/j.molcel.2015.01.017 (2015).

7 Feng, S. *et al.* Improved split fluorescent proteins for endogenous protein labeling. *Nat Commun* **8**, 370, doi:10.1038/s41467-017-00494-8 (2017).

8 Ran, F. A. *et al.* Genome engineering using the CRISPR-Cas9 system. *Nat Protoc* **8**, 2281-2308, doi:10.1038/nprot.2013.143 (2013).

9 Jonas, S., Weichenrieder, O. & Izaurralde, E. An unusual arrangement of two 14-3-3-like domains in the SMG5-SMG7 heterodimer is required for efficient nonsense-mediated mRNA decay. *Genes Dev* **27**, 211-225, doi:10.1101/gad.206672.112 (2013).

10 Zhang, P., Chen, X. B., Ding, B. Q., Liu, H. L. & He, T. Down-regulation of ABCE1 inhibits temozolomide resistance in glioma through the PI3K/Akt/NF-kappaB signaling pathway. *Biosci Rep* **38**, doi:10.1042/BSR20181711 (2018).

11 Kara, G., Tuncer, S., Turk, M. & Denkbas, E. B. Downregulation of ABCE1 via siRNA affects the sensitivity of A549 cells against chemotherapeutic agents. *Med Oncol* **32**, 103, doi:10.1007/s12032-015-0557-3 (2015).
