# Supplementary material for: DAP5 enables main ORF translation on mRNAs with structured and uORF-containing 5′ leaders
Source: Nat Commun. 2022 Dec 6;13:7510. doi: 10.1038/s41467-022-35019-5 (PMC9726905; doi:10.1038/s41467-022-35019-5)
Supplement: Supplementary file 3 — Description of Additional Supplementary Files [file 41467_2022_35019_MOESM3_ESM.pdf]

Description of Additional Supplementary Files

File Name: Supplementary Data 1
Description: Sequencing summary of RNA-Seq and Ribo Seq data in DAP5-null cells

File Name: Supplementary Data 2
Description: RocA and DAP5 common targets

File Name: Supplementary Data 3
Description: DAP5 and DENR common targets
