# Supplementary material for: DAP5 enables main ORF translation on mRNAs with structured and uORF-containing 5′ leaders
Source: Nat Commun. 2022 Dec 6;13:7510. doi: 10.1038/s41467-022-35019-5 (PMC9726905; doi:10.1038/s41467-022-35019-5)
Supplement: Supplementary file 7 — Reporting Summary [file 41467_2022_35019_MOESM7_ESM.pdf]

nature research

Corresponding author(s): Catia Igreja; Ramona Weber

Last updated by author(s): Nov 6, 2021

Reporting Summary

Nature Research wishes to improve the reproducibility of the work that we publish. This form provides structure for consistency and transparency in reporting. For further information on Nature Research policies, see our Editorial Policies and the Editorial Policy Checklist.

Statistics

For all statistical analyses, confirm that the following items are present in the figure legend, table legend, main text, or Methods section.

n/a	Confirmed	
☐	☒	The exact sample size (*n*) for each experimental group/condition, given as a discrete number and unit of measurement
☐	☒	A statement on whether measurements were taken from distinct samples or whether the same sample was measured repeatedly
☐	☒	The statistical test(s) used AND whether they are one- or two-sided<br>*Only common tests should be described solely by name; describe more complex techniques in the Methods section.*
☒	☐	A description of all covariates tested
☒	☐	A description of any assumptions or corrections, such as tests of normality and adjustment for multiple comparisons
☐	☒	A full description of the statistical parameters including central tendency (e.g. means) or other basic estimates (e.g. regression coefficient) AND variation (e.g. standard deviation) or associated estimates of uncertainty (e.g. confidence intervals)
☐	☒	For null hypothesis testing, the test statistic (e.g. *F*, *t*, *r*) with confidence intervals, effect sizes, degrees of freedom and *P* value noted<br>*Give P values as exact values whenever suitable.*
☒	☐	For Bayesian analysis, information on the choice of priors and Markov chain Monte Carlo settings
☒	☐	For hierarchical and complex designs, identification of the appropriate level for tests and full reporting of outcomes
☒	☐	Estimates of effect sizes (e.g. Cohen's *d*, Pearson's *r*), indicating how they were calculated

Our web collection on statistics for biologists contains articles on many of the points above.

Software and code

Policy information about availability of computer code

Data collection	*Provide a description of all commercial, open source and custom code used to collect the data in this study, specifying the version used OR state that no software was used.*
Data analysis	Integrative Genomics Viewer version 2.6.3,<br>Bowtie 2 version 2.2.9,<br>TopHat 2 version 2.1.1,<br>RiboTaper version 1.3.1,<br>QuasR, edgeR, goseq - R version 3.2.3<br>RiboDiff version 0.2.1,<br>CHOPCHOP,<br>Microsoft Excel<br>FlowJo (Becton Dickison)

For manuscripts utilizing custom algorithms or software that are central to the research but not yet described in published literature, software must be made available to editors and reviewers. We strongly encourage code deposition in a community repository (e.g. GitHub). See the Nature Research guidelines for submitting code & software for further information.

Data

Policy information about availability of data

All manuscripts must include a data availability statement. This statement should provide the following information, where applicable:

- Accession codes, unique identifiers, or web links for publicly available datasets
- A list of figures that have associated raw data
- A description of any restrictions on data availability

The data sets generated during this study are available at Gene Expression Omnibus (GEO: GSE155854). The source imaging data are available at Mendeley Data with the DOI: 10.17632/bzpfcnzg8w.1. Source Data are provided with this paper. Harringtonine, LTM and QTI datasets from human HEK293 cells were downloaded from the Sequence Read Archive database (accession: SRA056377, SRA160745). RocA and DENR datasets were retrieved from the GEO database accession numbers GSE70211 and GSE140084, respectively.

Field-specific reporting

Please select the one below that is the best fit for your research. If you are not sure, read the appropriate sections before making your selection.

☒ Life sciences ☐ Behavioural & social sciences ☐ Ecological, evolutionary & environmental sciences

For a reference copy of the document with all sections, see nature.com/documents/nr-reporting-summary-flat.pdf

Life sciences study design

All studies must disclose on these points even when the disclosure is negative.

Sample size	No statistical methods were used to establish sample size. The experiments were performed multiple times and were sufficient to obtain reproducible results.
Data exclusions	No Data were excluded
Replication	The manuscript contains only reproducible experiments and data. Each experiment was perform in three biological replicates, with the exception of RNA-Seq and Ribo-seq data sets where only 2 biological replicates were used. All the replications were successful.
Randomization	Not applicable. This study did not include data from human subjects or laboratory animals.
Blinding	No blinding was performed since the different experiments in the different cell types (WT and KO) or with the different reporters do not need to be blind. Each analysis was unbiased, performed in parallel and using the same measuring parameters.

Reporting for specific materials, systems and methods

We require information from authors about some types of materials, experimental systems and methods used in many studies. Here, indicate whether each material, system or method listed is relevant to your study. If you are not sure if a list item applies to your research, read the appropriate section before selecting a response.

Materials & experimental systems

n/a	Involved in the study	
☐	☒	Antibodies
☐	☒	Eukaryotic cell lines
☒	☐	Palaeontology and archaeology
☒	☐	Animals and other organisms
☒	☐	Human research participants
☒	☐	Clinical data
☒	☐	Dual use research of concern

Methods

n/a	Involved in the study	
☒	☐	ChIP-seq
☐	☒	Flow cytometry
☒	☐	MRI-based neuroimaging

Antibodies

Antibodies used

Antibodies used in this study:
Mouse monoclonal anti-GFP Roche Cat. #11814460001 1:3000
Rabbit polyclonal anti-HsWNK1 Cell Signaling Technology Cat. #4979 1:1000
Rabbit polyclonal anti-HsROCK1 Abcam Cat. #ab97592 1:1000
Mouse monoclonal anti-HsSHIP2 (INPPL1) Santa Cruz Biotechnology Cat. #sc-166641 1:1000
Rabbit polyclonal anti-HsDAP5 Bethyl laboratories Cat. #A302-249A-M 1:1000
Rabbit polyclonal anti-HseIF4A Abcam Cat. #ab31217 1:1000
Rabbit polyclonal anti-HseIF4A2 Abcam Cat. #ab31218 1:1000

Mouse monoclonal anti-HA (HRP) Roche Cat. #12013819001 1:5000
Mouse monoclonal anti-TUBULIN Sigma Aldrich Cat. #T6199 1:1000
Mouse monoclonal anti-V5 LSBio LifeSpan BioSciences, Inc. Cat. #LS-C57305 1:5000
Mouse monoclonal anti-Renilla luciferase Abcam Cat. #ab185925 1:1000
Rabbit polyclonal anti-HsABCE1 Abcam Cat. #ab32270 1:1000
Rabbit polyclonal anti-HseIF4E Bethyl laboratories Cat. #A301-154A 1:1000
Rabbit polyclonal anti-HseIF4G Bethyl laboratories Cat. #A301-776A 1:1000
Rabbit polyclonal anti-HsPABP Abcam Cat. #ab21060 1:3000
Donkey polyclonal anti-rabbit IgG (HRP) GE Healthcare Cat. #NA934V 1:10 000
Sheep polyclonal anti-mouse IgG (HRP) GE Healthcare Cat. #RPN4201 1:10 000

Validation

All antibodies are commercially available and validated. Information regarding validation and application can be found on the manufacturer's website.
anti-GFP Roche Cat. #11814460001 https://www.sigmaaldrich.com/deepweb/assets/sigmaaldrich/product/documents/294/951/11814460001bul.pdf
anti-HsWNK1 Cell Signaling Technology Cat. #4979 https://www.cellsignal.de/products/primary-antibodies/wnk1-antibody/4979
anti-HsROCK1 Abcam Cat. #ab97592 https://www.abcam.com/rock1-antibody-ab97592.html
anti-HsSHIP2 (INPPL1) Santa Cruz Biotechnology Cat. #sc-166641 https://datasheets.scbt.com/sc-166641.pdf
anti-HsDAP5 Bethyl laboratories Cat. #A302-249A-M https://www.fortislife.com/cms/files/A302-249A-1.pdf
anti-HseIF4A Abcam Cat. #ab31217 https://www.abcam.com/eif4a1-antibody-ab31217.html
anti-HseIF4A2 Abcam Cat. #ab31218 https://www.abcam.com/eif4a2-antibody-ab31218.html
anti-HA (HRP) Roche Cat. #12013819001 https://www.sigmaaldrich.com/deepweb/assets/sigmaaldrich/product/documents/760/007/12013819001bul.pdf
anti-TUBULIN Sigma Aldrich Cat. #T6199 https://www.sigmaaldrich.com/deepweb/assets/sigmaaldrich/product/documents/312/421/t6199dat.pdf
anti-V5 LSBio LifeSpan BioSciences, Inc. Cat. #LS-C57305 https://www.lsbio.com/antibodies/v5-tag-antibody-clone-sv5-pk1-elisa-flow-if-immunofluorescence-ihc-ip-wb-western-ls-c57305/57775
anti-Renilla luciferase Abcam Cat. #ab185925 https://www.abcam.com/renilla-luciferase-antibody-epr17791-ab185925.html
anti-HsABCE1 Abcam Cat. #ab32270 https://www.abcam.com/abce1-antibody-ab32270.html
anti-HseIF4E Bethyl laboratories Cat. #A301-154A https://www.fortislife.com/search?query=A301-154A&pageSize=15
anti-HseIF4G Bethyl laboratories Cat. #A301-776A https://www.fortislife.com/cms/files/A301-776A-1.pdf
anti-HsPABP Abcam Cat. #ab21060 https://www.abcam.com/pabp-antibody-ab21060.html
anti-rabbit IgG (HRP) GE Healthcare Cat. #NA934V https://www.fishersci.de/shop/products/anti-rabbit-igg-peroxidase-linked-species-specific-whole-antibody-from-donkey-secondary-antibody-cytiva/10379664
anti-mouse IgG (HRP) GE Healthcare Cat. #RPN4201 https://www.cytivalifesciences.com/en/us/shop/protein-analysis/immunoassays-biochemical-assays-accessories-and-reagents/reagents/amdex-enzyme-conjugates-for-elisa-antibody-reagents-p-00339#related-documents

Eukaryotic cell lines

Policy information about cell lines

Cell line source(s)	HEK293T cells were purchased from DSMZ (ACC 635)
Authentication	HEK293T cells were authenticated by DSMZ.
Mycoplasma contamination	HEK293T cells tested negative for mycoplasma contamination in the laboratory and by DSMZ.
Commonly misidentified lines (See ICLAC register)	No commonly misidentified cell lines were used in the study.

Flow Cytometry

Plots

Confirm that:

☒ The axis labels state the marker and fluorochrome used (e.g. CD4-FITC).

☒ The axis scales are clearly visible. Include numbers along axes only for bottom left plot of group (a 'group' is an analysis of identical markers).

☒ All plots are contour plots with outliers or pseudocolor plots.

☒ A numerical value for number of cells or percentage (with statistics) is provided.

Methodology

Sample preparation	HEK293T cells WT and DAP5 KO
Instrument	Becton Dickinson FACSMelody Cell Sorter
Software	FlowJo software (Becton Dickison)
Cell population abundance	HEK293T cells were analyzed based on the gating strategy described below.

Gating strategy	Preliminary FSC/SSC gates were performed for "living cells" without specific staining. After this selection, singlet gates on either BFP, GFP and mCherry were established. The boundaries between "positive" and "negative" staining cell populations were defined based on unstained control cells. In some figure panels, additional gating was done to determine the fraction of "GFP-positive"cells within the "BFP-positive" cell population.

☒ Tick this box to confirm that a figure exemplifying the gating strategy is provided in the Supplementary Information.
